# Perturbing Dynamin Reveals Potent Effects on the *Drosophila* Circadian Clock

**DOI:** 10.1371/journal.pone.0005235

**Published:** 2009-04-22

**Authors:** Valerie L. Kilman, Luoying Zhang, Rose-Anne Meissner, Elyssa Burg, Ravi Allada

**Affiliations:** 1 Department of Neurobiology and Physiology, Northwestern University, Evanston, Illinois, United States of America; 2 Center for Sleep and Circadian Biology, Northwestern University, Evanston, Illinois, United States of America; Yale School of Medicine, United States of America

## Abstract

**Background:**

Transcriptional feedback loops are central to circadian clock function. However, the role of neural activity and membrane events in molecular rhythms in the fruit fly *Drosophila* is unclear. To address this question, we expressed a temperature-sensitive, dominant negative allele of the fly homolog of *dynamin* called *shibire^ts1^* (*shi^ts1^*), an active component in membrane vesicle scission.

**Principal Findings:**

Broad expression in clock cells resulted in unexpectedly long, robust periods (>28 hours) comparable to perturbation of core clock components, suggesting an unappreciated role of membrane dynamics in setting period. Expression in the pacemaker lateral ventral neurons (LNv) was necessary and sufficient for this effect. Manipulation of other endocytic components exacerbated *shi^ts1^*'s behavioral effects, suggesting its mechanism is specific to endocytic regulation. PKA overexpression rescued period effects suggesting *shi^ts1^* may downregulate PKA pathways. Levels of the clock component PERIOD were reduced in the *shi^ts1^*-expressing pacemaker small LNv of flies held at a fully restrictive temperature (29°C). Less restrictive conditions (25°C) delayed cycling proportional to observed behavioral changes. Levels of the neuropeptide PIGMENT-DISPERSING FACTOR (PDF), the only known LNv neurotransmitter, were also reduced, but PERIOD cycling was still delayed in flies lacking PDF, implicating a PDF-independent process. Further, *shi^ts1^* expression in the eye also results in reduced PER protein and *per* and *vri* transcript levels, suggesting that *shibire*-dependent signaling extends to peripheral clocks. The level of nuclear CLK, transcriptional activator of many core clock genes, is also reduced in *shi^ts1^* flies, and *Clk* overexpression suppresses the period-altering effects of *shi^ts1^*.

**Conclusions:**

We propose that membrane protein turnover through endocytic regulation of PKA pathways modulates the core clock by altering CLK levels and/or activity. These results suggest an important role for membrane scission in setting circadian period.

## Introduction

Daily rhythms of sleep and wake are driven by transcriptional feedback loops in pacemaker neurons. In *Drosophila*, the transcription factor *Clock* (*Clk*) heterodimerizes with *cycle* (*cyc*) to directly activate key components of a principal feedback loop, *period* (*per*) and *timeless* (*tim*) [1], and of a secondary feedback loop, *par domain protein 1 (pdp-1)* and *vrille (vri)* [2], [3]. PER and perhaps TIM feed back and repress CLK/CYC DNA binding leading to molecular oscillations in clock components. VRI feeds back to repress transcription of *Clk* [3], while PDP may regulate clock output [4]. CLK also activates *clockwork orange (cwo)*, which represses CLK-activated transcription of its targets [5]–[7]. These molecular feedback loops are thought to operate in a cell-autonomous manner [8]. Several components of these feedback loops are conserved with mammals [1].

Molecular clocks are evident in many peripheral tissues, such as the eye, as well as the central brain [9]. Brain clocks are divided into 7 anatomical clusters: small and large ventral lateral neurons (sLNv, lLNv), dorsal lateral neurons (LNd), three groups of dorsal neurons (DN1, DN2, DN3), and the lateral posterior neurons (LPN) [10]–[14]. The neuropeptide Pigment Dispersing Factor (PDF) [15] is expressed uniquely by and is the only known transmitter of the LNv. Mutants of PDF or its receptor display short period damping rhythms [16]–[19]. *pdf^01^* pacemaker molecular oscillations are eventually low amplitude or phase-dispersed, indicating PDF feeds back to maintain synchrony [17], [20], [21]. Mammalian rhythms are also lost in mutants of the Vasoactive Intestinal Peptide (VIP) system [22], [23], indicating a conserved role for neuropeptidergic signaling in clocks. Under light-dark conditions (LD), the PDF+ sLNv mediate behavioral anticipation of the transition from dark to light (“morning”) while “evening” anticipation is mediated by PDF- clocks: the DN1, LNd, and one sLNv [24], [25]. Under constant darkness (DD), the LNv dominate behavioral period determination and reset non-PDF clocks [24], [26], [27]. PDF neurons may also receive a number of other neurotransmitter inputs [14], [28]–[33]. In addition, electrical silencing of PDF neurons suppresses core clock function [34], [35]. A number of intracellular signaling pathways have been identified as contributing to core circadian function [36]–[41]. However, the mechanisms of feedback between receptor and/or ion channel signaling and transcriptional feedback rhythms remain unclear.

To explore the role of the network in circadian function, we set out to perturb vesicle traffic as a way of disrupting intercellular communication. *shibire (shi)*, the *Drosophila* homolog of *dynamin*, is a GTPase necessary for vesicle scission [42], [43]. The dominant negative *shi^ts1^* allele [44], [45] has been used at the restrictive temperature (29°C) to inhibit synaptic transmission [45]. However *shi* is also involved in other endocytic pathways [46] that may affect intercellular signaling including receptor-mediated endocytosis and recycling of membrane proteins, such as ion channels. We show *shi^ts1^* expression in clock cells at 25°C results in robust long behavioral rhythms. Period effects are exacerbated by perturbing endocytic/endosomal pathways and suppressed by overexpressing *arrestin2* or a catalytic subunit of Protein Kinase A (PKA-C1). Long period results from PDF-independent delays in the molecular clock of the sLNv. With further impairment at 29°C, *shi^ts1^* expression in either the LNv or in peripheral eye clocks also drastically reduces *Clk* target gene levels. CLK itself is reduced in the sLNv and the long period is suppressed by *Clk* overexpression. These results suggest that modulation of cell membrane processes such as receptor signaling pathways may powerfully affect the molecular clock.

## Results

### *shibire^ts1^* Potently and Selectively Affects Circadian Period

**Figure 1:**
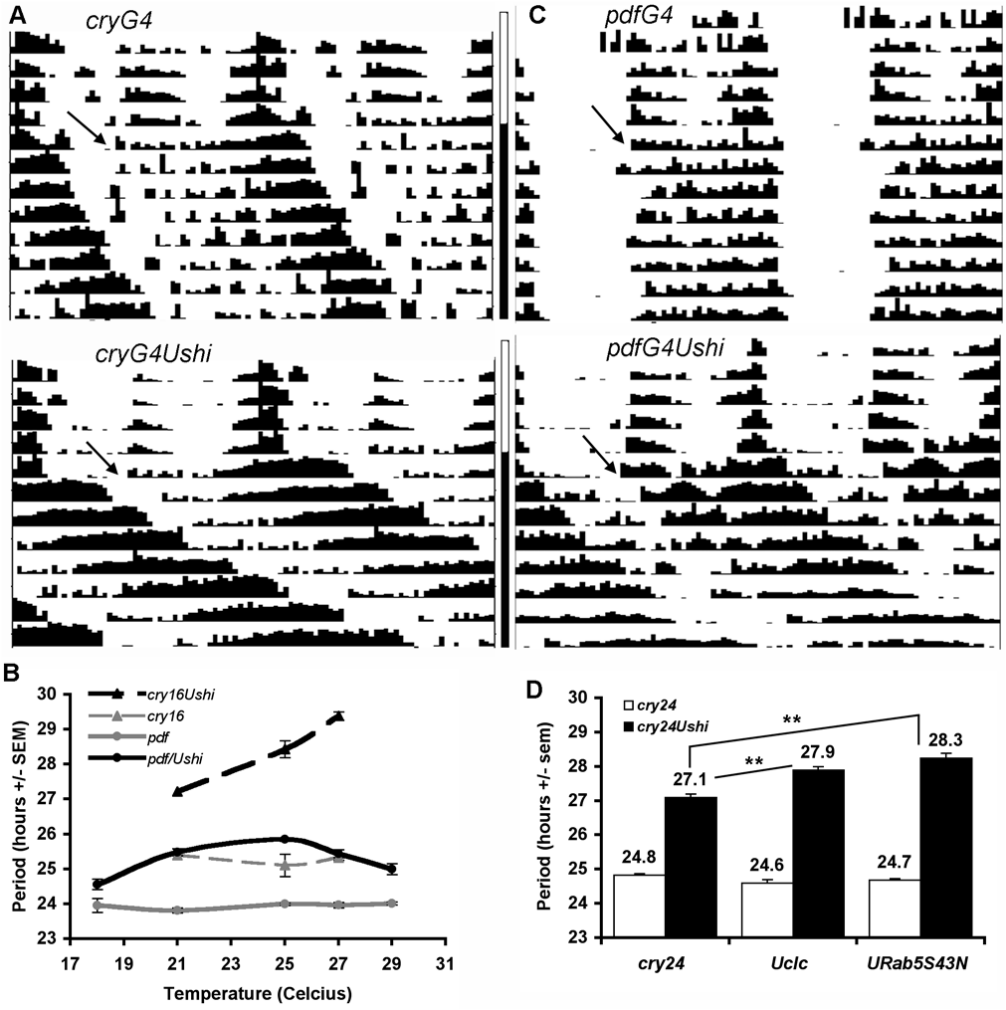
*shibire^ts1^* expression in pacemaker neurons lengthens period. A) Broad expression of *shibire^ts^* ^*1*^ including most circadian neurons induces long behavioral periods. Activity is plotted over two days on each horizontal line, with the second day repeated on the next line. The first four lines show behavior in LD. DD begins at the arrow in each graph. Relative to *cryGAL4-16*/+ controls, *cryGAL4-16/+*; *Ushi^ts1^/+* period is robust and up to 4 hours long (see table 1, table S1). B) Behavioral period is temperature sensitive in *shibire^ts^* ^*1*^-expressing flies. Driven by *cryGAL4-16*, period increases with temperature until flies become arrhythmic or die at 29°C. *pdfGAL4/+*; *Ushi^ts1^/+* behavioral period is about 1.8 hours longer than control at 25°C (see text). C) Activity plot of more restricted expression in PDF+ LNv at 25°C, (as in A). D) *shi^ts1^* effects on circadian period are modified by other endocytic components. Broad circadian expression (*cryGAL-24*; *Ushi^ts1^/+*) of clathrin light chain (*Uclc*) or a dominant negative version of a Rab5GTPase (*URab5S43N*), both involved in vesicle trafficking, have no effect on period themselves. However both exacerbate period-lengthening effects when expressed in combination with *shibire^ts^* ^*1*^. (**p<0.01).

To test the role of intercellular communication in the *Drosophila* circadian pacemaker, we expressed *shi^ts1^* throughout the pacemaker neuron system. Initial experiments utilized GAL4 lines, *crypGAL4-16 (cry16)* and *crypGAL4-24 (cry24)*, that drive expression throughout the key circadian neuronal groups (LN and DN, [14], [47]) and assayed behavior near the restrictive temperature at 29°C [45]. Under these conditions, most flies died or were arrhythmic consistent with the purported dominant negative function of this transgene (data not shown). As a result, we examined a range of semi-restrictive temperatures (21–27°C). Rather than weak, 24-hour rhythms, we found that flies exhibit robust circadian rhythms and their periods were strikingly long (Fig. 1A, 1B, Table 1, Table S1). In *cry16/UASshi^ts1^* flies, they were more than 4 hours longer than their GAL4 controls at 27°C (Figure 1B, Table S1). Rhythmicity was not compromised at these temperatures.

**Table 1:** Circadian Behavior Table.

Genotype	Period/SEM	Power/SEM	%R	n
*cryGAL4-16/+*	25.1+/−0.1	57.1+/−3.4	92	106
*cryGAL4-16/UASshi^ts1^*	28.4+/−0.1	103.9+/−5.9	93	91
*cryGAL4-24*	24.8+/−0.0	83.9+/−4.7	92	106
*cryGAL4-24;UASshi^ts1^/+*	27.1+/−0.1	105.0+/−5.2	98	100
*pdfGAL4/+*	24.0+/−0.0	73.2+/−5.5	90	72
*pdfGAL4/+;UASshi^ts1^/+*	25.8+/−0.0	74.8+/−5.2	92	93
*UASshi^ts1^/+*	23.9+/−0.0	82.7+/−5.3	88	93
*pdfGAL80/+;UASshi^ts1^/+*	24.0+/−0.1	51.1+/−6.6	74	43
*cryGAL4-24;pdfGAL80/+;UASshi^ts1^/+*	24.6+/−0.1	110.0+/−13.1	94	17
*pdfGAL4/+; p[Arr2]/+*	24.0+/−0.0	79.9+/−7.7	100	24
*pdfGAL4/+;UASshi^ts1^/p[Arr2]*	23.0+/−0.1	73.0+/−9.0	88	25
*pdfGAL4/UAS-PKA*	24.3+/−0.1	41.0+/−5.1	68	60
*pdfGAL4/ UAS-PKA;UASshi^ts1^/+*	24.8+/−0.1	60.2+/−10.4	78	27
*y w; pdf^01^*	23.3+/−0.7	4.2+/−0.9	23	52
*pdf^01^- UASshi^ts1^/+*	23.7+/−0.1	93.4+/−8.1	100	22
*cryGAL4-24; pdf^01^/+*	24.1+/−0.1	82.2+/−9.9	100	15
*cryGAL4-24; pdf^01^/ pdf^01^-Ushi^ts1^*	23.0+/−0.2	19.3+/−4.2	52	44
*pdfGAL4/+;pdf^01^/+*	23.6+/−0.1	63.9+/−12.1	88	8
*pdfGAL4/+; pdf^01^/ pdf^01^-Ushi^ts1^*	22.8+/−0.1	6.8+/−1.8	25	32
*pdfGAL4/+; UASClk/+*	23.1+/−0.1	72.7+/−8.5	96	24
*pdfGAL4/+; UASshi^ts1^/ UASClk*	23.3+/−0.1	90.4+/−7.4	95	43
*pdfGAL4/+; UASper/+*	24.0+/−0.1	20.4+/−3.6	66	38
*pdfGAL4/+; UASshi^ts1^/ UASper*	24.3+/−0.2	48.0+/−8.1	85	26
*pdfGAL4/+; UAStimII-5/+*	24.0+/−0.1	42.4+/−5.5	76	37
*pdfGAL4/+; UASshi^ts1^/ UAStimII-5*	24.2+/−0.1	81.5+/−8.8	87	38

Flies listed here were tested at 25°C. The average period+/−standard error of the mean (SEM) is listed, followed by the power of the rhythm (calculated as Power - Significance in Clocklab, Actimetrics)+/−SEM, % of flies rhythmic (P-S≥10), and n = total number of this genotype tested.

To map the neurons involved in this regulation, we expressed *shi^ts1^* only in the PDF+ pacemaker LNv. With expression driven by *pdfGAL4*, the period was nearly 2 hours longer than control, again with equivalent rhythmicity, at 25°C (Figure 1B, 1C, Table 1), indicating that *shi^ts1^* expression in PDF neurons is sufficient for robust period effects. Nonetheless, some of the *shi^ts1^* period effects may be derived from its expression in non-PDF neurons, since the period effects of *pdfGAL4*-driven *shi^ts1^* were less than those driven by *cry24* and *cry16*. We then expressed *shi^ts1^* broadly in all pacemaker neurons but blocked expression in the LNv using the GAL4 repressor GAL80 (Table 1, *cry24*; *pdfGAL80/+*; *UASshi^ts1^*/+, [24]). Period was not different from the GAL4 control, indicating that expression in PDF neurons is necessary and sufficient for period lengthening effects.

### *shi^ts1^* Lengthens Period by Disruption of an Endocytic Process

We found that *shi^ts1^* expression at 25°C induced strong period effects without affecting rhythmicity. These results do not appear to be due merely to *shi* overexpression as induction of different wild-type isoforms, singly and in combinations (long Δ0 with short Δ0, 2 independent short Δ2 isoforms, [48]), failed to cause significant period lengthening (data not shown). We also found that period difference was minimal when tested at 18°C, the nominally permissive temperature for this allele (Fig. 1B, Table 1). In contrast period differences from control when tested at 25°C were nearly equivalent whether flies were raised at 18°C or 21°C (data not shown). These results suggest that *shi^ts1^* functions primarily in adulthood to modulate period.

To determine whether the effects of *shi^ts1^* were through its role in endocytosis, we asked whether *shi^ts1^* period effects depend on the expression or activity of other known endocytic components. We first examined clathrin light chain, a principal component of the clathrin cage that surrounds certain endocytic vesicles [49]. Using the broad circadian driver *cry24*, expression of a clathrin light chain-GFP fusion alone did not affect period. However coexpression of *shi^ts1^* and *clc^GFP^* [50] resulted in significant period lengthening of almost an hour relative to *cry24*; *UASshi^ts1^/+* (Figure 1D). The fact that *clc^GFP^* enhances the long period of *cry24*; *UASshi^ts1^/+* suggests *shi^ts1^*'s specific effect on a clathrin-mediated endocytic process is responsible for its effect on period.

To assess the functional role of more downstream steps in the endocytic process, we expressed a dominant negative form of Rab5 (*Rab5S43N*; [51]), a GTPase that regulates vesicle traffic from the cell membrane into early endosomes [52]. Broad expression of *Rab5S43N* using the *cry24* driver failed to alter period or rhythmicity (Figure 1D; Table 1). In combination with *shi^ts1^* however (*cry24*; *UASshi^ts1^*/*UASRab5S43N*), period was significantly longer relative to *cry24*; *UASshi^ts1^*/+ (Figure 1D; Table 1). These data indicate that perturbing vesicles trafficked into early endosomes regulates circadian period.

We then manipulated arrestins, a clathrin-interacting protein involved in the downregulation, endosomal sorting, and signaling of a variety of G-protein coupled and growth factor receptor-initiated pathways [53]–[55]. We assayed flies carrying an extra genomic copy of *arrestin2* or *arr2* (p[w+; *arr2*]; gift of L. Zweibel), one of the fly “sensory” arrestins. Though it had no significant effects on circadian behavior by itself, p[w+; *arr2*] completely blocked period lengthening by *pdfGAL4/+*; *Ushi^ts1^*/+ (Table 1). This suggests overexpression of *arr2* can compensate for or prevent *shi^ts1^*'s effect. Taken together these data suggest the possibility that *shi^ts1^* affects rhythms by perturbing cell-surface initiated intracellular signaling pathways in the sLNv.

### PKA blocks period lengthening by *shi^ts1^*

Endocytosis and arrestin can modulate a variety of intracellular signals. Protein kinase A (PKA) regulates circadian behavior in flies [41] and has been shown to regulate clock output downstream of the clock [40]. To test if PKA played a role in *shi^ts1^*'s effects on period, we coexpressed PKA-C1, the catalytic subunit of PKA, with *shi^ts1^*. PKA alone in the LNv (*pdfGAL4/UASPKA*) did not appreciably alter period relative to control and slightly reduced rhythmicity (Table 1). In contrast, period in flies coexpressing PKA and *shi^ts1^* (*pdfGAL4/UASPKA*; *Ushi^ts1^*/+) was one hour shorter than in *pdfGAL4/+*; *Ushi^ts1^*/+ (Table 1; p<0.01). These data suggest that *shi^ts1^* may lengthen period by a mechanism that downregulates PKA.

### *shi^ts1^* Expression Delays PERIOD Rhythm Phase and Suppresses Rhythmic Amplitude

**Figure 2:**
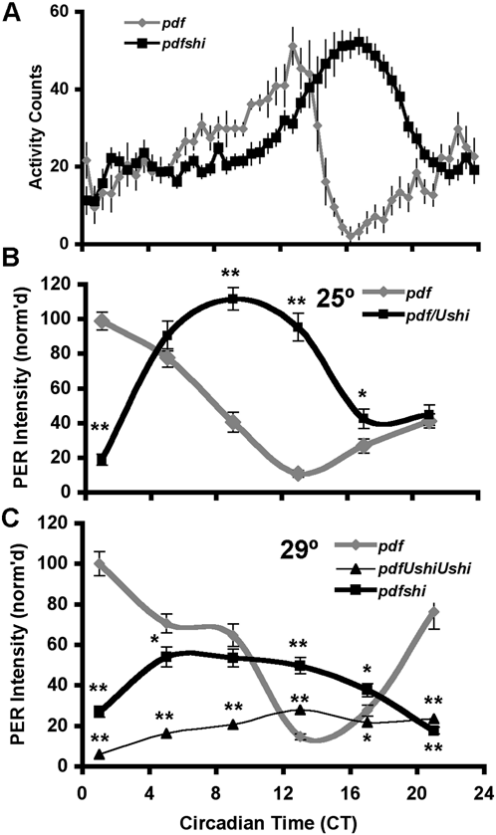
*shi^ts1^* expression in pacemaker neurons perturbs the molecular clock. A) Averaged activity of *pdfGAL4/+* and *pdfGAL4/+*; *Ushi^ts1^/+* flies on the third day of constant darkness (DD3). B) sLNv PER levels measured at timepoints throughout DD3. C) sLNv PER levels throughout DD3 in flies held at 29°C. Peak PER levels were reduced by *shi^ts1^* dose-dependently relative to the control. At both 29°C and 25°C, *pdfGAL4/+*; *Ushi^ts1^/+* PER cycling also appears delayed (*p<0.01, **p<0.05).

Because LNv expression of *shi^ts1^* is necessary and sufficient for behavioral period changes, we next focused on the cellular effects of *pdfGAL4*-driven *shi^ts1^* in the sLNv, crucial for setting period in DD conditions [25], [56]. We set out to examine sLNv PER cycling on the third day of constant dark conditions (DD3) following two days of LD entrainment at 25°C. We reasoned that this would allow us to more easily detect changes in PER cycling resulting from a 2-hour daily period change. To verify the magnitude of changes on DD3 we plotted the behavior of *shi^ts1^* expressing flies and their GAL4 controls on this day (Fig. 2A). Interestingly, both sets of flies show similar slow increases in activity but control activity then drops relatively abruptly. In the *pdfGAL4*/+ controls, activity level peaked at about CT13 followed by a rapid decline to a trough around CT17. The activity peak is broader in *shi^ts1^* expressing flies and the activity reduction is delayed several hours consistent with the observed behavioral period change. These results indicate that the PDF neuron output is intact as PDF neurons can still influence evening activity. Moreover, they suggest that *shi^ts1^* expressed in PDF neurons may specifically block termination rather than onset of evening activity.

Under the same conditions, (DD3, 25°C) PER staining in the sLNv cycles robustly in control flies (Figure 2B). In the *shi^ts1^*-expressing flies PER rhythms are phase-shifted relative to the control (both peak and trough occur later) and are apparently undamped, consistent with the behavior and with published reports of the critical role of the sLNv in setting DD period [25], [56]. Thus, not only does *shi^ts1^* alter behavioral period but also alters in a parallel manner the rhythms of the core molecular oscillator in the master pacemaker sLNv.

*UASshi^ts1^* is temperature-sensitive and has been used to disrupt synaptic transmission in specific neurons at 29°C [45]. Though the average behavioral period of *pdfGAL4/+*; *Ushi^ts1^/+* flies at 29°C was not longer than at 25°C (Fig. 1B, Table 1, Table S1), the standard error was high (Table S1); some *shi^ts1^*-driven periods were so short as to be outside the range of controls. When we examined PER cycling at 29°C, the sLNv on DD3 were delayed relative to controls, similar to the case at 25°C (Fig. 2C). However in addition peak PER levels were substantially reduced. Increases in *shi^ts1^* dose resulted in further peak PER reductions (Figure 2C, *pdfGAL4/+*; *Ushi^ts1^/ Ushi^ts1^*). Peak reductions at 29°C are likely due to reduced cycling amplitude rather than desynchrony among cells as we observed no evidence of desynchronized nuclear entry among the sLNv, and standard error for PER staining intensity was comparable to (*pdfGAL4/+*; *Ushi^ts1^/+*, p = 0.29) or lower than (*pdfGAL4/+*; *Ushi^ts1^/ Ushi^ts1^*, p<0.05) control. In addition though the strength of the behavioral rhythm was reduced (Table S1), we saw no evidence of complex rhythms. Thus *shi^ts1^* appears to lengthen period by perturbing the core clock without appreciably affecting molecular synchrony among the sLNv, suggesting that PDF neuron outputs are intact.

### PDF is Required for Behavioral Output, not for Delay of the sLNv Molecular Clock

PDF is the only known neurotransmitter output of the core LNv pacemaker cells and is thought to mediate synchrony among the sLNv and between pacemaker groups [16], [21]. We hypothesized that *shi^ts1^* may modulate PDF signaling and in turn, affect PDF feedback, thus affecting circadian period. To test this model, we assayed the effects of expressing *shi^ts1^* in a *pdf^01^* mutant background. Both *cry24*; *UASshi^ts1^-pdf^01^/pdf^01^* and *pdfGAL4/+*; *UASshi^ts1^-pdf^01^/pdf^01^* flies exhibited weak short period (∼23 hour) rhythms, comparable to *y w*; *pdf^01^* mutants and completely distinct from the longer periods observed in *shi^ts1^* expressing PDF-positive flies (Table 1). These results indicate that the behavioral effects of *shi^ts1^* require PDF.

**Figure 3:**
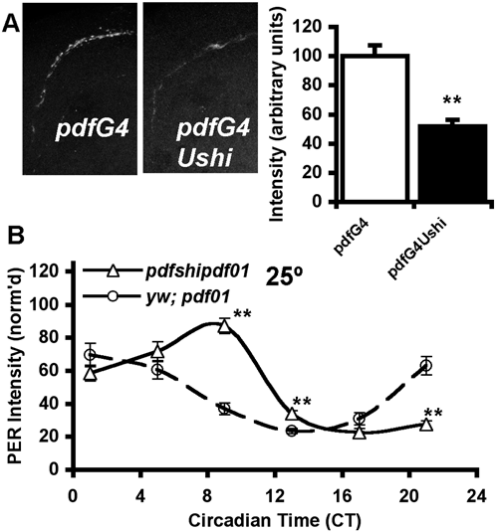
*shibire^ts1^* effects on PER rhythms are independent of PDF. A) *shibire^ts1^* reduces PDF levels. PDF levels were measured in sLNv terminals of the dorsal protocerebrum on DD3 (25°C). Overall PDF levels were lower in *pdfGAL4/+*; *Ushi^ts1^/+* (**p<0.05, see methods). B) On DD3 (25°C), sLNv PER was relatively normal in *y w*; *pdf^01^* flies. *shi^ts1^* expression in the absence of PDF (*pdfGAL4/+*; *Ushi^ts1^- pdf^01^/ pdf^01^*) results in PER delays similar to PDF+ flies (*p<0.01, **p<0.05).

Some studies show that PDF levels in the dorsal brain terminals of the sLNv cycle in a circadian fashion [57] (but see also [58]) and are altered by chronic manipulations of electrical activity in the circuit [17], [35], [59]–[61]. These findings suggest changes in neuronal activity may affect PDF levels in the dorsal terminals, perhaps by regulating PDF release. We measured PDF levels in the sLNv terminals as a crude measure of potential changes in PDF signaling and release. PDF levels in *pdfGAL4/+*; *Ushi^ts1^/+* flies were consistently lower than in *pdfGAL4/+* (Fig. 3A, see methods). The combined data show a significant reduction in the level of PDF in the sLNv terminals.

We then asked if PDF is required for *shi^ts1^* effects on the molecular clock in the sLNv. To assay the state of the molecular clock, flies lacking PDF were collected at six timepoints on DD3 as before. To our surprise, sLNv PER cycling in *pdfGAL4*; *UASshi^ts1^-pdf^01^/pdf^01^* was delayed relative to *y w*; *pdf^01^* (Figure 3B), an effect similar to the delay of *pdfGAL4/+*; *UASshi^ts1^*/+ relative to *pdfGAL4*/+ (Figure 2B). Peak and trough were both delayed. They results show that PDF is required to convey the altered molecular oscillations to behavioral outputs. More significantly, these results unambiguously demonstrate that PDF is dispensable for *shi^ts1^* effects on the core molecular oscillator of the sLNv.

### Peripheral Clocks are Also Strongly Affected by *shi^ts1^* Expression

**Figure 4:**
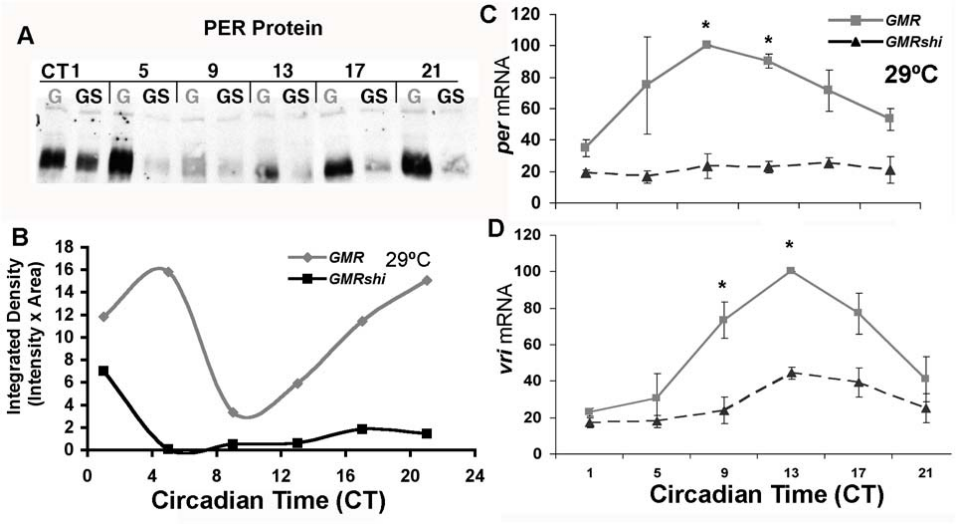
*shi^ts1^* expression in the eye reduces clock gene products. Flies entrained at 29°C were collected on DD1. A) Whole head PER Westerns, dominated by PER in the eye, showed PER in control heads peaking at CT5 and reaching trough soon after. In contrast, *shi^ts1^* expression in the eye (*GMRGAL4/+*; *Ushi^ts1^/+*) greatly reduced PER levels in DD. (G = *GMRGAL4/+*, GS = *GMRGAL4/+*; *Ushi/+*). B) Quantification of A). C), D) Core clock gene transcripts are reduced in *shi^ts1^*-expressing flies. RT-PCR for *per* and *vri* (C, D respectively, flies as in A) reveal sharp reductions in transcript levels (*p<0.05).

Though PDF signaling does not contribute to the molecular effect of *shi^ts1^*, period lengthening may occur through perturbation of other intercellular signals specific to core pacemakers. To determine if *shi^ts1^*'s effects are specific to the network of circadian neurons that drive locomotor rhythms, we expressed *shi^ts1^* instead in the eye. The majority of PER in the head is derived from peripheral clocks in the eye that cycle coherently in LD cycles but damp gradually in DD [62]. We expressed *shi^ts1^* in all photoreceptors using *GMRGAL4*. Flies raised at 18°C were shifted to 29°C at the beginning of a two-day LD entrainment period. We examined whole-head PER levels throughout DD1 as peripheral cycling remains appreciable at this point. Cycling in the control *GMRGAL4/+* flies was robust, peaking at CT5 and showing roughly 3-fold amplitude (Fig. 4A, 4B). In contrast, PER in *GMRGAL4/+*; *UASshi^ts1^*/+ heads was lower at the beginning of DD and quickly fell to low baseline levels, remaining relatively stable throughout DD1. This dramatic effect on PER levels was similar to that seen in the sLNv of *pdfGAL4/+*; *Ushi^ts1^/ Ushi^ts1^* flies at 29°C (Fig. 2C), indicating that *shi^ts1^*'s effects are not specific to networked pacemaker clocks.

Reduced PER could be due to increased turnover or reduced synthesis. We asked if reduced PER levels were a reflection of reduced mRNA levels. Fly heads expressing *shi^ts1^* in the eye were collected as before on DD1 and analyzed for *per* mRNA levels. *per* in controls cycled robustly, peaking at CT9 with roughly 2.5-fold amplitude. By contrast *per* in *GMRGAL4/+*; *UASshi^ts1^*/+ was low and stable throughout the day (Fig. 4C). These effects were not specific to *per*, as *vri* levels from the same flies were also substantially reduced (Fig. 4D). These results favor the interpretation that *shi^ts1^* perturbs the clock by reducing transcription of at least two CLK-activated core clock components, *per* and *vri*, and that this leads to reductions in PER levels.

### CLK is Reduced in *shi^ts1^*-expressing Pacemaker sLNv

**Figure 5:**
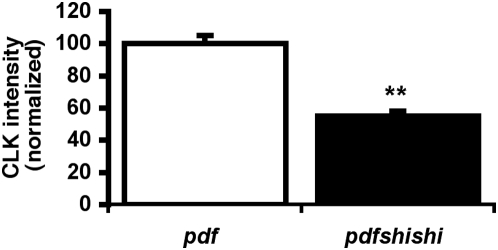
CLK levels are reduced in *shibire^ts1^*-expressing sLNv. *pdfGAL4/+*; *Ushi^ts1^/ Ushi^ts1^* flies were entrained at 29°C. On DD3 (CT16), sLNv nuclear CLK levels were reduced to about 60% of control levels. (**p<0.05).

It is notable that *per* and *vri* are both direct targets of the transcriptional activator CLK [2], [63], [64]. One mechanism by which both mRNAs could be reduced coordinately is through reductions of CLK-mediated transcription. To assess whether *shi^ts1^* might affect CLK, we quantified CLK levels in the sLNv of flies expressing high doses of *shi^ts1^* (*pdfGAL4/+*; *Ushi^ts1^/ Ushi^ts1^*) at 29°C, conditions under which PER was stably reduced 5-fold from the control peak (Fig. 2C). Under the same conditions, we found nuclear CLK in the sLNv is also reduced to approximately 60% of the control level (Fig. 5). Thus it is plausible that CLK transcription is reduced by *shi^ts1^*, leading to reductions in *per* and other clock transcripts. If so, we might expect that artificially increasing CLK levels could mitigate period lengthening in *shi^ts1^*-expressing flies. We coexpressed *Clk* with *shi^ts1^* and assayed behavioral rhythms. Flies expressing *Clk* in the PDF+ LNv show behavioral rhythms approximately one hour shorter than the GAL4 control but of equivalent rhythmicity (Table 1). Coexpression of *Clk* and *shi^ts1^* resulted in nearly identical behavior to expression of *Clk* alone; i.e. about one hour short. Thus *Clk* expression in the LNv either compensated for or completely blocked the effect of *shi^ts1^* on behavioral rhythms. Interestingly, coexpression of the CLK targets *per* or *tim* with *shi^ts1^* was also able to return period to control levels (Table 1), supporting the interpretation that *shi^ts1^* modulates period through *Clk*'s transcriptional activation of *per* and *tim*.

Taken together these data suggest that *shi^ts1^* may affect clocks by reducing CLK levels and/or transcriptional activity and slowing feedback in the molecular clock. As our work indicates that *shi^ts1^*'s effects operate by compromising endocytic pathways, we suggest that membrane events leading to PKA signaling may modulate the clock by regulating expression, turnover, and/or activity of CLK.

## Discussion

Our data suggest an important function for membrane events, specifically endocytosis, in circadian timing. While previous studies have demonstrated roles for neural activity in circadian output [59], [60], in sustaining molecular rhythms [34], and in synchrony [21], [35], [56], [61], our work strongly suggests a substantial role in circadian timing. Expression of *shi^ts1^* in pacemaker neurons results in strikingly long periods, suggesting potent effects on circadian timing through perturbing vesicle scission. These effects are enhanced by co-expression of other components of endocytic pathways leading to early endosomes, consistent with *shi* function in traffic, recycling, and turnover of cell membrane components. PKA expression rescues period defects induced by *shi^ts1^*, suggesting a functional link between the membrane, PKA, and behavioral period. The LNv-expressed *shi^ts1^* results in delays in the phase of PER molecular rhythms in the sLNv sufficient to account for the delay in behavior. While *shi^ts1^* effects on behavior require PDF, those on the molecular clock of the sLNv are PDF-independent, implicating a novel pathway. In fact these perturbations of the molecular clock are not specific to locomotor pacemakers, but appear in peripheral clocks as well, suggesting membrane-clock interactions are a general property of clock cells. Reductions in the levels of CLK and CLK-activated transcripts are consistent with the hypothesis that membrane events regulate the molecular clock through CLK.

The importance of a clock component can be inferred by the magnitude of its phenotype when perturbed. By this criterion, the magnitude of the *shi^ts1^* period effects argues for a critical role for endocytosis in setting circadian period. *shi^ts1^* overexpression can lead to period changes over three hours longer than controls at 25°C. Indeed, the observed period effects are comparable to or even greater than those when overexpressing *per*, *tim* or *Clk*, suggesting that perturbation of cell membrane recycling may be as important as these canonical core clock elements to period determination [47], 65.

*shi^ts1^* effects are modulated by components of endocytic pathways. Coexpression of a *clathrin light chain-GFP* (*clc-GFP*) or a dominant negative form of *Rab5* results in ∼1 hour lengthening of *shi^ts1^* period effects and overexpression of wild-type *arr2* fully rescues *shi^ts1^* period lengthening. Given that we predict that *shi^ts1^* blocks endocytosis, the enhancement of effects by CLC seems paradoxical. One possibility is that overexpression of these components titrates away key endocytic components and thus further enhances the *shi^ts1^* effects. Alternatively, as we co-expressed a CLC-GFP fusion, this chimeric protein may not be fully functional and thus may impair vesicle scission further. The period enhancing effects of the dominant negative form of Rab5 suggest an important role for endosomal intermediates, while rescue by *arr2* or PKA suggests involvement of intracellular signaling pathways. At least two genes involved in endocytosis, *syndapin* and *beta-adaptin* (*Bap*), have been shown to be rhythmic by microarray studies [66]. Of note, syndapin is thought to directly interact with dynamin [67]. Rhythms of endocytosis could reflect or even amplify rhythms of intercellular communication. In addition, intracellular signaling pathways induced by these inputs could alter the intracellular oscillator. Taken together, rhythmic regulation of endocytic pathways may define a novel feedback loop in the circadian clockwork.

Potent effects of *shi^ts1^* are evident by reductions in CLK expression and/or function. Expression of *shi^ts1^* in the eye results in reductions in CLK-activated transcripts, *per* and *vri*. Similarly, reduced PER levels are also evident in *shi^ts1^* expressing sLNv. These reductions in CLK-activated genes are accompanied by reductions in CLK levels in the sLNv. Prior studies have shown that CLK expression and activity is primarily regulated posttranscriptionally [68], [69]. PER is thought to deliver DBT as well as other kinases to CLK resulting in its phosphorylation, reducing its half-life and transcriptional activation [70]. In addition, a number of kinases have been implicated as CLK regulators, most notably PKA which was shown to upregulate CLK-CYC transcription in cultured cells [39]. PKA is activated by cAMP. Interestingly, mutants in the cAMP dependent phosphodiesterase *dunce* that show increased peak cAMP levels also show short period behavior [41]. Notably, we find that inhibition of PKA in the LNv mimics *shi^ts1^* with lengthened periods and reduced PER levels (L. Zhang and R. Allada, unpublished data). We propose that *shi^ts1^* expression may modulate these cAMP pathways to alter CLK phosphorylation, suppressing its activity and/or destabilizing it, ultimately leading to long period behavior.

Several lines of evidence indicate that *shi^ts1^* effects are not operating principally by blocking pacemaker neural output. Expression of tetanus toxin in PDF neurons blocks responses to arousing effects of cocaine, indicating that PDF neurons use a classical neurotransmitter and that tetanus toxin is expressed at functional levels capable of blocking this process [71]. Yet tetanus toxin expression in PDF+ cells does not significantly alter period or rhythmicity [72]. In *shi^ts1^* expressing flies, delayed PDF neuronal clocks still delay the offset of evening behavior, implying PDF cells can still reset evening clocks. In addition, we did not observe desynchronization of molecular rhythms among the sLNv as might be expected if communication were disrupted. Period altered *shi^ts1^*-expressing flies also largely preserve rhythmicity at 25°C suggesting a primary clock effect rather than an output effect. Likewise PDF, the only known sLNv output, is also not necessary for *shi^ts1^* molecular effects. In *pdf^01^* mutants, *shi^ts1^* expression blocks the effects on behavioral period but does not block the effect of *shi^ts1^* on PER LNv rhythms. The uncoupling of sLNv molecular rhythms from behavioral rhythms clearly demonstrates an output function for PDF in pacemaker neuron function. This also implies that other neural clusters drive behavior in *pdf^01^*. Moreover, these results demonstrate that *shi^ts1^*'s effects on sLNv PER do not operate through PDF. Taken together these data suggest the period differences we see do not result primarily from alterations of sLNv transmitter output. Instead it seems likely *shi^ts^* perturbs another target or pathway regulating sLNv activity.

While effects of *shi^ts1^* are typically tested at 29°C or above, *shi^ts1^* effects noted here have been observed at just 25°C, below the reported paralytic temperature for *shi^ts1^* [73], [74]. However, ultrastructural *shi^ts1^* effects have been observed even at the nominal permissive temperature (18°C) for behavioral paralysis [74]. Thus, *shi^ts1^* is likely modestly defective at 18°C and this impairment grows with increasing temperature until a threshold is reached at which paralysis is evident when driven in motorneurons. However under conditions of overexpression, the temperature threshold for various phenotypes may differ from paralysis. Our finding of slight period lengthening relative to controls even at 18°C is consistent with a modest defect, with core clock effects getting stronger gradually as the temperature increases. Our evidence that *shi^ts1^* is not perturbing outputs (at least at 25°C) raises that possibility that other membrane scission-sensitive processes, such as receptor endocytosis, may have a lower threshold for disruption than synaptic transmission.

What might be the nature of the membrane perturbation evoked by *shi^ts1^*? More broadly, endocytosis regulates membrane protein turnover, and a variety of targets could influence neuronal activity, including ion channels, pumps, and transporters, which in turn could feedback to regulate the core clock. Endocytosis has a well-established role in down-regulation of metabotropic or ionotropic receptors. In the sLNv, potential receptors include (but are not limited to) acetylcholine, GABA, serotonin, dopamine, histamine ([28]–[30]), and neuropeptides such as IPNamide [14]. Ion channel density may also be modulated by endocytosis [75]–[77] and could influence core clock rhythms [34], [59], [60]. On the other hand, our finding that PKA overexpression can suppress *shi^ts1^* effects on period provide evidence that down regulation of G-protein coupled receptors that stimulate cAMP and PKA may be a mechanism for *shi* action. The identification and functional analysis of the relevant membrane targets of *shi^ts1^* will be critical to understanding the role of the membrane in circadian function.

## Materials and Methods

### Behavior

Crosses were maintained at 21°C until behavior. Male flies were then entrained at 25°C (unless otherwise indicated) for 2 or 5 days in 12-hour light∶12 hour dark conditions before release into constant darkness. Activity was monitored with the Trikinetics DAM system. LD activity averages were compiled with in-house DCM software. DD behavior was analyzed using Clocklab software (Actimetrics). Period differences were evaluated with *t-test*.

### Immunostaining

Flies were entrained for 2–3 days in LD before release into DD. On the third day of DD flies were collected and 6–8 were dissected at the indicated timepoints. Brain tissue was fixed 40 minutes in 4% formalin in phosphate buffer (PB). Primary rabbit anti-PER (sometimes embryo pre-absorbed) at 1∶4000 diluted in PBT (PB with 0.3% Triton) with 10% normal serum was applied overnight. A secondary goat anti-rabbit Alexa 594 antibody (Molecular Probes) was applied at 1∶600 overnight. Final washes in PBT and PB were followed by mounting in 80% glycerol/PB. For experiments with PDF-positive genotypes, tissue was double-labeled for PER and PDF to aid cell identification. Mouse anti-PDF (1∶800, DSHB #C7) and 1∶500 goat anti-mouse Alexa 488 (Molecular Probes) were applied with the PER primary and secondary, respectively. For CLK staining, guinea pig anti-CLK [78] at 1∶10000 was applied with mouse anti-PDF as above. Secondary detection used goat anti-guinea pig Alexa 647 and goat anti-mouse 488 (1∶600).

### Quantitation

Slides were coded to allow blind imaging, data collection, and analysis. Experiments were run at least twice and in each experiment staining from 4–8 hemispheres/condition was imaged on a Nikon C1 confocal (PER and PDF, 60× oil objective, PDF terminals 20×, 1 µm steps). PER, CLK quantitation: Cells were identified by position, size, and PDF double-labeling when applicable. The entire soma (nucleus only for CLK) was manually circled in the single confocal section of maximum diameter and digitally quantified using ImageJ (NIH). A nearby non-stained region was quantified for background subtraction. Average intensity for each cell was used to compute a mean for the group. Experiments were repeated at least twice and results were consistent. Subsequently data from individual cells was scaled to the typical peak control average for each experiment (CT1 of *pdfG4/+* in figure 2; in figure 3 CT9 of *pdfGAL4/+*; *Ushi-pdf^01^/pdf^01^*. PDF quantitation: The terminal segment of sLNv neurites in the dorsal brain was quantified as a single number representing the mean intensity of pixels on a hand-drawn line in ImageJ. Measurements were averaged across genotype within four timepoints for each replication. PDF levels in were lower in *UASshi^ts1^*-expressing flies at nearly all timepoints. However as two replications of the experiment a) gave inconsistent fluctuations and b) did not reflect control PDF oscillations reported in the literature, we combined data across timepoints within genotype for final means+/−SEM seen in graphs. Averaged data were compared using *t-test*.

### Flies

Fly stocks were obtained from the following sources: M. Rosbash (*cry16*, *cry24*, *pdfGAL4*, *pdfGAL80*, *pdf^01^*); T. Kitamoto (*UASshi^ts1^*); I. Mellman (*UASclc*); M. Gonzalez-Gaitan (*UASRab5S43N*); L. Zweibel (p[arr2]); M. Ramaswami (*UASTf82-1*; *UASTf27-25* and *UASTf114-5* and *UASTf114-2*); D. Kalderon (*UAS-PKAC1*). All other fly stocks were obtained from Bloomington Stock Center.

### Westerns

Crosses were raised at 18°C. Western blotting was performed as reported elsewhere [79]. After two days of entrainment at 29°C, heads from entrained flies were collected on dry ice at four-hour interval timepoints throughout DD1. Protein extracts were made from 20–30 heads (same within experiment) and run on a 6% SDS-PAGE gel. After Ponceau staining confirmed equal loading, nitrocellulose blots were probed with 1∶10000 rabbit anti-PER followed by 1∶2000 HRP anti-rabbit. Signal was detected with ECL following kit directions (Amersham). Autoradiographs were scanned and quantified using NIH ImageJ.

#### Quantitative real-time PCR

Flies were collected as for Westerns. Total RNA was isolated from frozen whole heads using InVitrogen's TRIzol reagent and manufacturer's protocol. DNA was removed from RNA extracts using RQ1 DNase from Promega (5 µl DNase in 120 µl total reaction volume). DNase was removed from RNA extracts using the following method: 120 µl of phenol∶chloroform∶isoamyl alcohol (25∶24∶1 from Amresco) was added to each sample. Samples were shaken by hand for 1 minute and centrifuged at top speed at room temperature for 5 minutes. 100 µl of the aqueous top phase was transferred to a new tube. 10 µl of 3M sodium acetate and 300 µl of ethanol were added to each sample and incubated at −70°C for 1 hour. Samples were spun down at top speed at 4°C for 15 minutes. Supernatants were removed and the pellet washed with 1 ml 75% ethanol. Samples were spun down at top speed at 4°C for 15 minutes. Supernatants were removed, and the pellet was dried upside-down for 5–10 minutes. RNA pellets were dissolved in 30–50 µl water. Real-time PCR reactions were run using the Applied Biosystems 7900HT fast real-time PCR instrument. Data were collected using SDS software v2.2.1. Data were analyzed using the 2^−DeltaDeltaCt^ method [80] using *RP49* expression values to normalize for differences in RNA amount among samples. For PCR reactions, ∼100 ng RNA were used per reaction. Total reaction volume was 10 µl and reactions were run in 384-well plates. Primer sets (forward, reverse respectively) are as follows: *per* (5′-CAGCAGCAGCCTAATCG-3′, 5′-GAGTCGGACACCTTGG-3′), *vri* (5′-TGTTTTTTGCCGCTTCGGTCA-3′, 5′-TTACGACACCAAACGATCGA-3′), *RP49* (5′-CGACGCTTCAAGGGACAGTATC-3′, 5′-TCCGACCAGGTTACAAGAACTCTC-3′). PCR cycling parameters were as follows: 30 minutes at 50°C, 15 minutes at 95°C, and 30 cycles of 15 seconds at 94°C, 30 seconds at 55°C, and 30 seconds at 72°C.

## Supporting Information

Table S1 Temperature-dependent behavior effects. Table arranged as Table 1.
(0.03 MB DOC)
