# Supplementary material for: Perturbing Dynamin Reveals Potent Effects on the Drosophila Circadian Clock
Source: PLoS One. 2009 Apr 22;4(4):e5235. doi: 10.1371/journal.pone.0005235 (PMC2668759; doi:10.1371/journal.pone.0005235)
Supplement: Table S1 — Temperature-dependent behavior effects. Table arranged as Table 1. (0.03 MB DOC) [file pone.0005235.s001.doc]

| **Genotype** | **Temp** | **Period/SEM** | **Power/SEM** | **%R** | **n** |
| --- | --- | --- | --- | --- | --- |
| *pdfGAL4/+* | **18ºC** | 24.0+/-0.2 | 55.8+/-4.3 | 96 | 44 |
| *pdfGAL4/+;Ushi^ts1^/+* |  | 24.6+/-0.1 | 59.5+/-5.9 | 94 | 46 |
|  |  |  |  |  |  |
| *cryGAL4-16/+* | **21ºC** | 25.4+/-0.3 | 41.8+/-7.0 | 66 | 41 |
| *cry16GAL4/Ushi^ts1^* |  | 27.2+/-0.2 | 45.9+/-7.7 | 68 | 37 |
| *pdfGAL4/+* |  | 23.8+/-0.1 | 37.6+/-5.6 | 79 | 28 |
| *pdfGAL4/+;Ushi^ts1^/+* |  | 25.5+/-0.1 | 56.7+/-6.7 | 97 | 32 |
|  |  |  |  |  |  |
| *cryGAL4-16/+* | **27ºC** | 25.3+/-0.1 | 57.3+/-6.1 | 87 | 37 |
| *cry16GAL4/Ushi^ts1^* |  | 29.4+/-0.2 | 86.8+/-10.3 | 86 | 29 |
| *pdfGAL4/+* |  | 24.0+/-0.1 | 58.2+/-7.3 | 79 | 34 |
| *pdfGAL4/+;Ushi^ts1^/+* |  | 25.4+/-0.1 | 56.9+/-5.7 | 87 | 37 |
|  |  |  |  |  |  |
| *pdfGAL4/+* | **29ºC** | 24.0+/-0.0 | 84.1+/-4.5 | 100 | 66 |
| *pdfGAL4/+;Ushi^ts1^/+* |  | 25.0+/-0.2 | 54.7+/-4.6 | 85 | 66 |
